# Home-use agents in the treatment of dentin hypersensitivity: clinical effectiveness evaluation with different measurement methods

**DOI:** 10.1007/s00784-025-06155-1

**Published:** 2025-01-15

**Authors:** Gizem Ayan, Tuğba Mіsіllі, Mehmet Buldur

**Affiliations:** Faculty of Dentistry, Department of Restorative Dentistry, Çanakkale Onsekiz Mart University, Çanakkale, 17100 Turkey

**Keywords:** Arginine, CPP-ACP, Dentin hypersensitivity, Novamin, Potassium nitrate, Propolis

## Abstract

**Objectives:**

This study aimed to evaluate the effectiveness of home-use desensitizing agents over an 8-week period by comparing them using different measurement methods.

**Methods:**

A randomized, controlled clinical trial was conducted with 180 individuals aged between 18 and 70 who clinically diagnosed dentin hypersensitivity (DH) in two or more non-adjacent teeth. Subjects who met the inclusion criteria (*n* = 164) were randomly allocated into five test groups—using Casein phosphopeptide-amorphous calcium phosphate (CPP-ACP), Arginine, Novamin, Propolis, and Potassium nitrate—and a control group using standard fluoride toothpaste. The status of DH was assessed at week 4 and week 8 by the same independent examiner. Changes from baseline in Dentine Hypersensitivity Experience Questionnaire-15 (DHEQ-15), Schiff Sensitivity Scale (SSS) and Visual Analog Scale (VAS) were analysed using ANOVA and Kruskall-Wallis tests.

**Results:**

All test groups showed statistically significant improvements in DH at weeks 4 and 8 compared to baseline in the DHEQ-15, VAS, and SSS assessments (*p* < 0.005). In the control group, significant improvements were observed only in the VAS and SSS measurements from baseline to weeks 8 (*p* < 0.005). The CPP-ACP group demonstrated the greatest reduction in scores by the end of week 8 compared to baseline, with DHEQ-15 (56.68 ± 17.87), VAS (6.52 ± 1.48), and SSS (2.32 ± 0.56).

**Conclusions:**

Among the tested agents, the CPP-ACP group demonstrated the most notable reduction in DH symptoms by week 8, highlighting its potential as an effective method for alleviating DH symptoms in a home-use agents.

**Clinical relevance:**

Home-use desensitizing agents are effective in the treatment of DH, improving the daily activities of patients who cannot access clinical care and ensuring the relief of DH before clinical invasive procedures.

**Trial registration:**

ClinicalTrials.gov Identifier: NCT06216262.

## Introduction

Dentin hypersensitivity (DH) is defined as a condition characterized by a short, sharp pain in response to thermal, tactile, osmotic, or chemical stimuli, which cannot be attributed to any other dental defect or pathology [1]. The prevalence of DH in the general population has been reported to range between 8% and 57% [2]. In individuals with periodontal disease, this rate ranges from 72 to 98% [3]. DH is explained by the hydrodynamic theory, which is based on the movement of fluid within dentinal tubules in both directions and the activation of intratubular nerves or mechanoreceptors on the pulp surface [4].

The treatment of DH is generally achieved through the combined use of home-use and office-based desensitizing treatments. However, due to its non-invasive nature, it has been recommended to start with home-use methods for treatment [5]. Home-use treatment methods include the application of toothpastes, gels, and mouthwashes by patients. These materials exert their effect on sensitive teeth through two simple mechanisms: desensitization of the nerves and occlusion of the exposed dentinal tubules [6].

Arginine is a natural amino acid present in saliva that, in combination with calcium carbonate and phosphate ions, forms plugs within dentinal tubules [7]. Novamin (calcium sodium phosphosilicate), when introduced into the oral cavity, causes sodium ions to replace hydrogen ions, leading to the release of calcium-phosphate ions. These minerals precipitate in the dentinal tubules, resulting in the occlusion of the tubules [8]. Propolis is known as a DH-relieving agent with antimicrobial, antiviral, antifungal, and antioxidant properties, and it has a tubule-blocking effect [9]. Casein phosphopeptide-amorphous calcium phosphate (CPP-ACP) is a milk-derived phosphoprotein that prevents DH by occluding dentinal tubules through the deposition of protein and calcium/phosphate ions [10]. Potassium nitrate (KNO_3_) is used both in mouthwash and toothpaste forms as an agent that blocks nerve transmission. In both forms, potassium nitrate has been reported to have a therapeutic effect in alleviating DH [11]. These agents can be effectively used in DH treatment by either occluding dentin tubules through various mechanisms or inhibiting nerve transmission.

The Dentin Hypersensitivity Experience Questionnaire (DHEQ), developed by Boiko in 2010, is one of the indices that assesses Oral Health-Related Quality of Life (OHRQoL) [12]. The DHEQ is a scale consisting of 48 questions [13]. The DHEQ-15 is a shorter form of the DHEQ that is less burdensome for researchers and participants compared to the long form. It increases response rates, reduces data collection costs, and facilitates its use among older individuals or larger populations [14].

This study aimed to comprehensively examine the effectiveness of home-use desensitizing agents in DH treatment by evaluating them with the Turkish version of the DHEQ-15 [15], Visual Analog Scale (VAS), and Schiff Sensitivity Scale (SSS), and assessed the changes in their effectiveness over time. Additionally, it sought to evaluate the DHEQ-15 scale in the Turkish population and assessed its applicability in different demographic groups in DH treatment. The null hypothesis (H₀) of this study was that there would be no difference in the effectiveness of home-use desensitizing agents in alleviating dentin hypersensitivity.

## Materials and methods

This study was conducted between January and July 2024 at the Department of Restorative Dentistry, Faculty of Dentistry, Çanakkale Onsekiz Mart University. It was approved by the Çanakkale Onsekiz Mart University Clinical Research Ethics Committee on May 3, 2023, with decision number 2023/07–14. This randomized clinical trial followed the recommendations of the Consolidated Standards of Reporting Trials (CONSORT) [16]. The clinical trial is registered with NCT06216262, and the registration date is January 19, 2024.

Sample size calculation was performed using the G*Power 3.1 software (Heinrich-Heine-Universität Düsseldorf, Düsseldorf, Germany), and total sample size was calculated as 144 with an alpha-type error of 0.05, a power (1-beta) of 0.85, and an effect size of 0.3287 (medium effect size) obtained from a previous similar study [17].

**Fig. 1:**
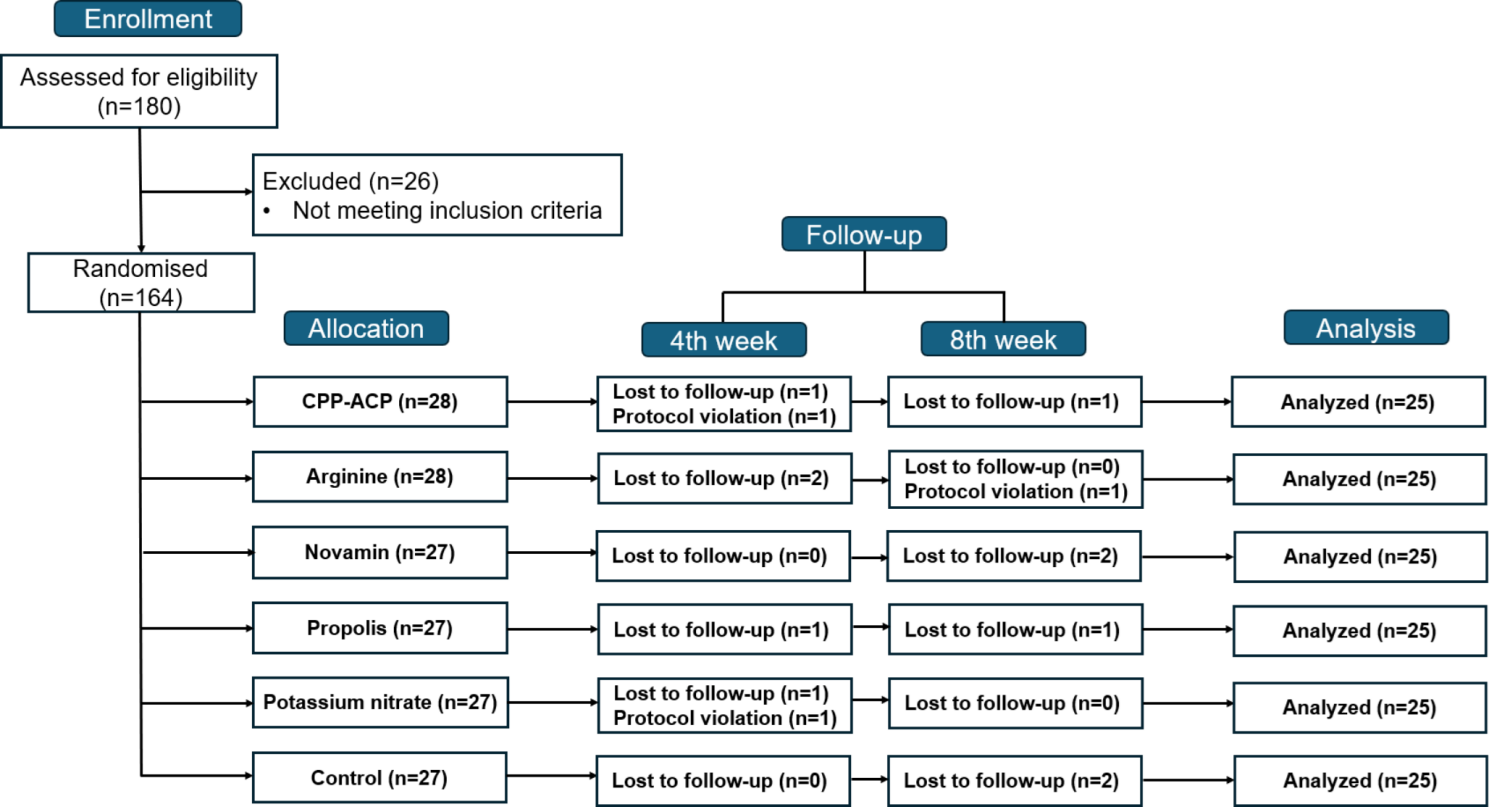
Diagram of patient enrollment

Figure 1 summarizes the participant progress throughout the study, from enrollment to analysis. Initially, 180 individuals aged 18–70 with sensitivity in two or more non-adjacent teeth were assessed for eligibility. Pregnant or breastfeeding women, individuals with advanced periodontal disease, chronic conditions that could affect the study results, major oral pathologies, use of anticonvulsants, antihistamines, antidepressants, sedatives, or tranquilizers, use of desensitizing toothpaste in the past 3 months, and individuals who have undergone periodontal disease treatment in the past 12 months were excluded from the study. Individuals with suspected pulpitis, cavities, cracked enamel, or teeth supporting removable partial dentures were also excluded from the study. 26 participants were excluded for not meeting the inclusion criteria, leaving 164 eligible participants who were then randomized into six groups. Participants were provided with necessary information both in writing and verbally, and informed consent forms were signed voluntarily before the study commenced.

The groups and active ingredients are presented in Table 1. Participants in the first group were provided with a toothpaste containing CPP-ACP (GC Tooth Mousse; GC America, Alsip, IL, USA), the second group received a toothpaste containing Arginine (Colgate Sensitive Pro-relief Toothpaste; Colgate Palmolive, Swidnica, Poland), the third group received a toothpaste containing Novamin (Sensodyne Repair&Protect Toothpaste; GlaxoSmithKline (GSK), Waterford, Ireland), the fourth group received a toothpaste with Propolis (Eyup Sabri Tuncer Natural Propolis Extract Toothpaste; Eyup Sabri Tuncer, Istanbul, Turkey), the fifth group was given a mouthwash containing Potassium nitrate (Sensodyne Cool Mint Mouthwash; GlaxoSmithKline (GSK), Maidenhead, Berkshire, UK), and the sixth group, as the control group, received a standard fluoride toothpaste (Colgate Total Toothpaste; Colgate Palmolive, Guangzhou, China). All participants were provided with soft toothbrushes (Colgate 360° Soft Toothbrush; Colgate Palmolive, Guangzhou, China) and were instructed to use only these toothbrushes.

Participants in the Arginine, Novamin, Propolis, and Control groups were instructed to brush their teeth twice daily, for 2 min each time, for 8 weeks. Participants in the CPP-ACP group were instructed to apply the toothpaste to their tooth surfaces with their finger for 3 min every night, after brushing with standard fluoride toothpaste. Participants were instructed not to spit after applying the toothpaste and to avoid eating or drinking for at least 30 min. Participants in the Potassium nitrate group were instructed to rinse with the mouthwash twice daily, in the morning and evening, after brushing with standard fluoride toothpaste. Participants were instructed to fill the cap up to the 10 ml line, rinse the mouth with the mouthwash for 1 min, and then spit it out. They were also informed that they should not rinse their mouth with water after using the mouthwash.

Standardized usage has been ensured at every stage of the treatment process. All patients were provided with written and verbal application instructions specific to their groups in a consistent manner. They were advised to avoid acidic foods and drinks, as well as the frequent consumption of sugary foods. Additionally, they were educated on brushing their teeth using the Modified Stillman technique without applying excessive force. They were informed that tooth brushing should be done with a soft toothbrush 30 min after breakfast and dinner.

Participants were evaluated for DH at baseline, as well as at the 4th and 8th weeks. During follow-up assessments, 5 participants missed their follow-up visit and 2 had protocol violations by the 4th week, while by the 8th week, 6 participants missed their follow-up visit and 1 had a protocol violation. Ultimately, 25 participants from each group were included in the final analysis (Fig. 1).

The Turkish version of the DHEQ-15 [15], a 7-point Likert scale ranging from ‘strongly agree’ to ‘strongly disagree’, was used to evaluate DH. Scores for all the questions on the form were rated from 1 to 7, with the total DHEQ-15 score calculated to range between 15 and 105 [18].

To determine the individual’s pain score for DH, the VAS was used, and they were asked to rate their pain severity on a scale from 0 to 10, where 0 represents no pain and 10 represents unbearable pain [19].

Two non-adjacent test teeth with the highest sensitivity were identified for each individual. For each test tooth, a single dentist conducted a clinical examination and applied air to the cervical area of the tooth from a distance of 1 cm for 1 s using an air spray. The SSS was then determined separately for each tooth. Based on the patient’s response to the air stimulus, a restorative dentist assigned a score between 0 and 3 for each tooth. 0: No response; 1: Response to the air stimulus is present, no need to stop the stimulus; 2: Response to the air stimulus is present, stimulus should be stopped; 3: Response to the air stimulus, considers the stimulus painful [20].

The average score was calculated by summing the SSS of both test teeth and dividing the total by two. The effectiveness of the home-use desensitizing agents in treating DH was evaluated using DHEQ-15, VAS, and SSS, with scores compared at baseline, 4 weeks, and 8 weeks.

**Table 1:** Groups and active ingredients

Desensitizing agents	No.of participants	Products	Active Ingredients
CPP-ACP	28	GC Tooth Mousse	Pure water, Glycerol, CPP-ACP, D-Sorbitol, Silicon Dioxide, CMC-Na, Propylene glycol, Titanium dioxide, Xylitol, Phosphoric acid, Guar gum, Zinc Oxide, Sodium Saccharin, Ethyl p-hydroxybenzoate, Magnesium oxide, Butyl p-hydroxybenzoate, Propyl p-hydroxybenzoate.
Arginine	28	Colgate Sensitive Pro Relief Toothpaste	Pro-Argin with 8.0% arginine and 1450 ppm fluorides as sodium monofluorophosphate in calcium carbonate base.
Novamin	27	Sensodyne Repair and Protect Toothpaste	Calcium sodium phosphosilicate (NOVAMIN) 5.0% w/w, sodium fluoride (0.2299% w/w) (fluoride 0.104% w/w), aroma, carbomer, cocamidopropyl betaine, glycerin, hydrated silica, PEG-8, sodium methyl cocoyl taurate, sodium saccharin, titanium dioxide.
Propolis	27	Eyup Sabri Tuncer Natural Propolis Extract Toothpaste	Sorbitol, Aqua, Calcium Carbonate, Hydrated Silica, Glycerin, Xylitol, Polysorbate 20, Cocamidopropyl Betaine, Disodium Phosphate, Xanthan Gum, Aroma, Phenylpropanol, Caprylyl Glycol, Stevia Rebaudiana Extract, Propolis Extract, Mentha Piperta Oil, Menthol, Thymus Vulgaris Leaf Oil.
Potassium nitrate	27	Sensodyne Cool Mint Mouthwash	Aqua, Glycerin, Sorbitol, Potassium nitrate, PEG-60 Hydrogenated Castor Oil, Poloxamer 407, Sodium Benzoate, Aroma, Disodium Phosphate, Methylparaben, Propylparaben, Sodium Phosphate, Sodium Fluoride, Sodium Saccharin, CI 42,090. Contains: 3% w/w Potassium nitrate and 0.048% w/w Sodium Fluoride (217ppm Fluoride).
Control	27	Colgate Total Toothpaste	Sodium fluoride, sodium monofluorophosphate, dicalcium phosphate with 1450 ppm fluorides.

### Statistical analysis

The reliability for the DHEQ-15 scale was tested using Cronbach’s alpha. The normality assumption was tested using the Shapiro-Wilk test, variance homogeneity was tested using Levene’s test, and sphericity assumption was tested using Mauchly’s W test. ANOVA was used for comparing groups with normal distribution, and the Kruskal-Wallis test was used when normal distribution was not present. For each desensitizing agent group, changes over time were examined using Repeated Measures ANOVA (Greenhouse-Geisser statistic) when the normality assumption was met, and the Friedman test when normal distribution was not present. To identify the group or groups responsible for the differences, Post Hoc Bonferroni and Modified Bonferroni tests were performed. The analyses were conducted using Statistical software (SPSS 25.0; IBM, Armonk, NY, US). A p-value of < 0.05 was considered statistically significant.

## Results

The study comprised 150 participants, with 93 females (62%) and 57 males (38%). The ages of the participants ranged from 19 to 70, with a mean [± SD] age of 40.9 [± 15.1]. The most commonly affected teeth were ranked, with the lower premolar teeth being the most frequent, accounting for 20.3%. The distrubution of affected teeth was as follows upper premolars (18.3%), lower anterior teeth (16.7%), upper molars (12.3%), upper canines (10.4%), upper anterior teeth (8.6%), lower molars (8%), and lower canines (5.3%). The educational levels of the participants were: 37.3% university, 23.3% master’s degree, 22.7% high school, 8.7% primary education, 6% secondary education, and 2% doctora.

The results of the reliability analysis conducted to test the consistency of the applied DHEQ-15 scale based on the responses of the participants are provided in Table 2. Accordingly, the high Cronbach’s alpha reliability coefficients calculated for the desensitizing agent groups tested at all time points (baseline, 4 weeks, and 8 weeks) indicate that the scale was highly reliable.

**Table 2:** Reliability analysis of DHEQ-15 scores based on desensitizing agents and evaluation periods

	Reliability (α)		
Desensitizing agents	Baseline	4th week	8th week
CPP-ACP	0.901	0.962	0.957
Arginine	0.843	0.941	0.962
Novamin	0.923	0.941	0.974
Propolis	0.895	0.977	0.986
Potassium nitrate	0.897	0.971	0.966
Control	0.902	0.953	0.960

α: Cronbach’s α > *0.7*, acceptable reliability

The distributions of DHEQ-15 scores by desensitizing agent and measurement times are provided in Table 3. In the control group, no statistically significant difference was observed in scale scores over time (*p* = 0.068), whereas statistically significant differences were found in scale scores over time in all other groups (*p* < 0.001). For all desensitizing agents, a significant decrease was observed at the 4th week compared to baseline (except for Arginine with *p* = 0.017 and Propolis with *p* = 0.014; *p* < 0.001 for all other groups). However, a significiant decrease between the 4th and 8th weeks was found only in the CPP-ACP and Arginine groups (*p* = 0.022 for CPP-ACP; *p* = 0.033 for Arginine). When considering different desensitizing agents within each assessment period, no differences were found in baseline scale scores among the agents. However, statistically significant differences were observed at the 4th and 8th weeks (*p* < 0.001). According to the Bonferroni tests conducted for the 8th week, all tested agent groups exhibited statistically significantly lower DHEQ-15 scores compared to the control group (*p* < 0.001 for CPP-ACP; *p* = 0.003 for Potassium nitrate; *p* = 0.008 for Arginine; *p* = 0.009 for Novamin; *p* = 0.021 for Propolis). This was only valid for the CPP-ACP and Potassium nitrate groups at the 4th week (*p* < 0.001 and *p* = 0.013, respectively).

**Table 3:** Dentin hypersensitivity experience Questionnaire-15 (DHEQ-15) scores by evaluation periods and desensitizing agents

	Baseline		4th week		8th week		Between evaluation periods	
Desensitizing agents	Min-Max	Mean ± SD (Median)	Min-Max	Mean ± SD (Median)	Min-Max	Mean ± SD (Median)	Test Statistic	*p*
CPP-ACP	48–105	77.84 ± 16.39 (80)^**z**^	15–85	35.60 ± 18.92 (27)^**A, y**^	15–50	21.16 ± 10.71 (15)^**A, x**^	47.574	**< 0.001**
Arginine	54–97	77.84 ± 13.84 (75)^**z**^	15–82	49.44 ± 20.77 (48)^**AB, y**^	15–82	37.52 ± 21.85 (35)^**A, x**^	31.618	**< 0.001**
Novamin	31–104	72.68 ± 19.56 (75)^**y**^	15–104	51.44 ± 20.14 (52)^**AB, x**^	15–104	39.16 ± 24.59 (33)^**A, x**^	38.786	**< 0.001**
Propolis	56–105	77.68 ± 15.11 (76)^**y**^	15–105	51.64 ± 27.49 (50)^**AB, x**^	15–105	41.56 ± 31.46 (27)^**A, x**^	24.700	**< 0.001**
Potassium nitrate	41–99	74.6 ± 15.44 (77)^**y**^	15–86	46.40 ± 25.12 (47)^**A, x**^	15–85	36.36 ± 21.99 (33)^**A, x**^	41.953	**< 0.001**
Control	49–105	82.36 ± 17.99 (88)	30–105	70.48 ± 23.34 (74)^**B**^	15–103	68.72 ± 25.5 (74)^**B**^	5.379	0.068
Between agents	**Test Statistic**	***p***	**Test Statistic**	***p***	**Test Statistic**	***p***		
	4.265	0.512	25.729	**< 0.001**	37.406	**< 0.001**		

Kruskal-Wallis test for agent comparisons; Friedman test for time comparisons (*p* < 0.05)

x-z: Different lowercase letters indicate statistically significant differences in the same row

A-B: Different uppercase letters indicate statistically significant differences in the same column

The distributions of SSS scores by desensitizing agent and measurement times are shown in Table 4. Statistically significant time-related differences were found in SSS scores for all groups, including the control group (*p* < 0.001). For all desensitizing agents, the values at the 4th and 8th weeks were statistically similar and significantly lower compared to baseline values (*p* < 0.001 for all groups). In the control group, only the 8th week scores were found to be significantly lower than the baseline values (*p* = 0.027). When considering different desensitizing agents within each assessment period, no differences were found in baseline SSS scores among the agents (*p* = 0.235). However, statistically significant differences were observed at the 4th and 8th weeks (*p* < 0.001). According to the Bonferroni tests for the 4th and 8th weeks, the SSS values for all agent groups were significantly lower compared to the control group (*p* < 0.001 for CPP-ACP; *p* < 0.001 for Potassium nitrate; *p* < 0.001 for Arginine; *p* = 0.009 and *p* = 0.001 for Novamin, respectively; *p* = 0.002 and *p* = 0.007 for Propolis, respectively). Additionally, at the 4th week, the CPP-ACP group’s score was lower than the Novamin group’s score (*p* = 0.041).

**Table 4:** Schiff Sensitivity Scale (SSS) scores by evaluation periods and desensitizing agents

	Baseline		4th week		8th week		Between evaluation periods	
Desensitizing agents	Min-Max	Mean ± SD (Median)	Min-Max	Mean ± SD (Median)	Min-Max	Mean ± SD (Median)	Test Statistic	*p*
CPP-ACP	1.5-3	2.5 ± 0.52 (2.5)^**y**^	0–1	0.36 ± 0.37 (0.50)^**A, x**^	0–1	0.18 ± 0.32 (0)^**A, x**^	446.927	**< 0.001**
**Arginine**	1–3	2.28 ± 0.61 (2)^**y**^	0–3	0.82 ± 0.84 (0.50)^**AB, x**^	0–3	0.56 ± 0.85 (0.5)^**A, x**^	42.769	**< 0.001**
**Novamin**	1–3	2.12 ± 0.56 (2)^**y**^	0–3	1 ± 0.71 (1)^**B, x**^	0–3	0.68 ± 0.79 (0.5)^**A, x**^	40.765	**< 0.001**
Propolis	1–3	2.22 ± 0.56 (2)^**y**^	0–3	1.02 ± 1.02 (1)^**AB, x**^	0–3	0.88 ± 0.98 (0.5)^**A, x**^	38.174	**< 0.001**
**Potassium nitrate**	1.5-3	2.36 ± 0.55 (2)^**y**^	0–3	0.84 ± 0.77 (1)^**AB, x**^	0–3	0.62 ± 0.75 (0.5)^**A, x**^	44.667	**< 0.001**
Control	1–3	2.38 ± 0.56 (2.5)^**y**^	0.5-3	2.02 ± 0.71 (2)^**C, xy**^	0–3	1.9 ± 0.79 (2)^**B, x**^	19.514	**< 0.001**
Between agents	**Test Statistic**	***p***	**Test Statistic**	***p***	**Test Statistic**	***p***		
	6.812	0.235	44.408	**< 0.001**	45.371	**< 0.001**		

Kruskal-Wallis test for agent comparisons; Friedman test for time comparisons (*p* < 0.05)

x-y: Different lowercase letters indicate statistically significant differences in the same row

A-C: Different uppercase letters indicate statistically significant differences in the same column

The distributions of VAS scores by desensitizing agent and measurement times are shown in Table 5. Statistically significant time-related differences were found in VAS scores for all groups (*p* < 0.001). A significant decrease was observed at the 4th week compared to baseline for all groups, including the control group (except for Propolis with *p* = 0.007, *p* < 0.001 for all other groups). However, a significiant decrease between the 4th and 8th weeks was found only in the CPP-ACP, Potassium nitrate, and Control groups (*p* = 0.022 for CPP-ACP; *p* = 0.004 for Potassium nitrate; *p* = 0.039 for control). When considering different desensitizing agents within each assessment period, no differences were found in baseline VAS scores among the agents (*p* = 0.411). However, statistically significant differences were observed at the 4th and 8th weeks (*p* < 0.001). Propolis showed higher VAS scores than CPP-ACP at both the 4th and 8th weeks (*p* = 0.031 and *p* = 0.029, respectively), while Potassium nitrate showed higher VAS scores than CPP-ACP only at the 8th week (*p* = 0.047). The control group generally recorded the highest VAS scores among the analyzed groups.

**Fig. 2:**
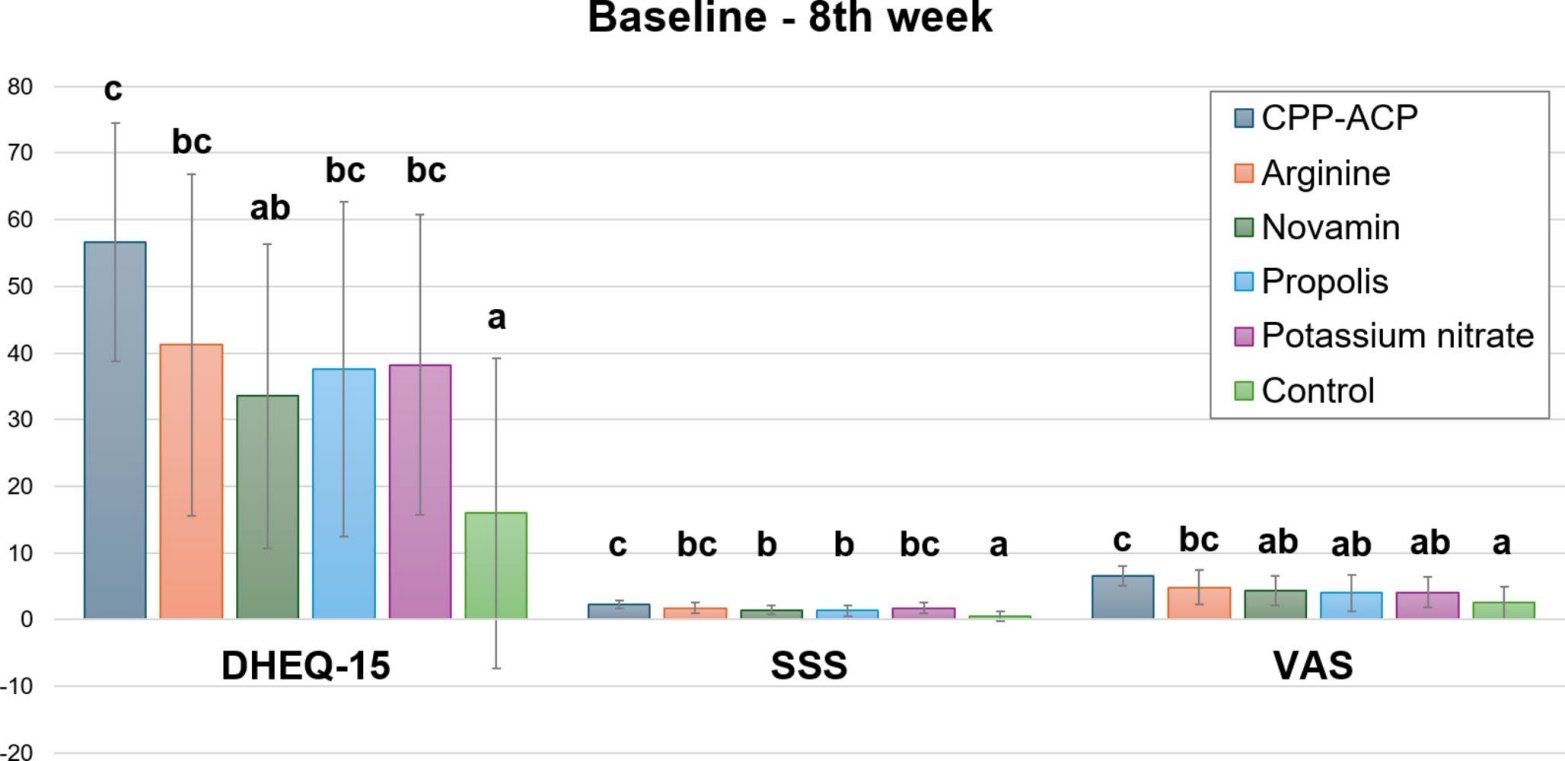
Changes in DHEQ-15, SSS, and VAS scores from baseline to 8th week according to desensitizing agents. *(Kruskal-Wallis test*,* p* < 0.05)

Figure 2 illustrates the changes in DHEQ-15, SSS, and VAS scores from baseline to the 8th week according to desensitizing agents. Statistically significant differences were found among the desensitizing agents in the changes of scores from baseline to the 8th week for all measures (*p* < 0.001). According to the Bonferroni tests, the highest DHEQ-15 change among all desensitizing agents was observed in the CPP-ACP group, which only led to a significant difference compared to Novamin (*p* = 0.030). Except for Novamin, all agents showed a significant higher change compared to the control group (*p* < 0.001 for CPP-ACP; 0.005 for Arginine; 0.046 for Propolis; 0.022 for Potassium nitrate). When examining changes in SSS scores, the values for the CPP-ACP group were found to be significantly higher than those for the Novamin (*p* = 0.008) and Propolis (*p* = 0.002) groups. Additionally, all agents showed greater changes compared to the control group (*p* = 0.008 for Novamin; *p* = 0.037 for Propolis, *p* < 0.001 for all other groups). When considering VAS scores, all desensitizing agents showed higher score changes compared to the control group, but this resulted in a significant difference only in the CPP-ACP and Arginine groups (*p* < 0.001; *p* = 0.027). On the other hand, the CPP-ACP group exhibited statistically significantly higher change values compared to Potassium nitrate, Novamin, and Propolis (*p* = 0.007; *p* = 0.030; *p* = 0.009, respectively).

**Table 5:** Visual Analog Scale (VAS) scores by evaluation periods and desensitizing agents

	Baseline		4th week		8th week		Between evaluation periods	
Desensitizing agents	Min-Max	Mean ± SD (Median)	Min-Max	Mean ± SD (Median)	Min-Max	Mean ± SD (Median)	Test Statistic	*p*
CPP-ACP	6–10	7.44 ± 1.19 (7)^**z**^	0–5	2.52 ± 1.48 (3)^**A, y**^	0–4	0.92 ± 1.26 (0)^A,**x**^	47.574	**< 0.001**
Arginine	4–10	7.36 ± 1.52 (8)^**y**^	0–10	3.84 ± 2.46 (3)^**ABC, x**^	0–10	2.52 ± 2.69 (2)^A**B, x**^	41.407	**< 0.001**
Novamin	5–10	6.84 ± 1.55 (6)^**y**^	0–10	3.56 ± 2.10 (3)^**AB, x**^	0–10	2.52 ± 2.42 (2)^A**B, x**^	40.923	**< 0.001**
Propolis	5–10	7.2 ± 1.5 (7)^**y**^	0–10	4.44 ± 2.81 (4)^**B, x**^	0–10	3.2 ± 3.11 (3)^BC, x^	37.324	**< 0.001**
Potassium nitrate	4–10	6.84 ± 1.57 (7)^**z**^	0–8	3.44 ± 1.96 (3)^**AB, y**^	0–8	2.76 ± 2.07 (3)^**BC, x**^	68.455†	**< 0.001**
Control	4–10	7.4 ± 1.78 (7)^**z**^	2–9	5.52 ± 1.92 (5)^**C, y**^	0–9	4.96 ± 2.21 (5)^**C, x**^	22.364†	**< 0.001**
Between agents	**Test Statistic**	***p***	**Test Statistic**	***p***	**Test Statistic**	***p***		
	5.041	0.411	5.499‡	**< 0.001**	35.223	**< 0.001**		

ANOVA and Kruskal-Wallis tests for agent comparisons; Repeated Measures ANOVA and Friedman tests for time comparisons

*p* < 0.05, ‡: ANOVA test, †: Repeated Measures ANOVA test

*x-z: Different lowercase letters indicate statistically significant differences in the same row*

*A-C: Different uppercase letters indicate statistically significant differences in the same column*

## Discussion

The null hypothesis (H₀) of this study, stating that “there would be no difference in the effectiveness of home-use desensitizing agents in alleviating DH,” has been rejected. Although previous randomized controlled clinical trials have investigated the effectiveness of home-use desensitizing agents in separate studies, this research provides an approach that compares the effectiveness of five different home-use desensitizing agents—Arginine, Novamin, Propolis, Potassium nitrate, and CPP-ACP—with a standard fluoride toothpaste used in the control group. The study has a relatively large sample size and a substantial number of participants who completed the study, which enhances the validity of the results.

DH is the exposure of dentinal tubules due to gingival recession along with cementum loss and/or enamel loss, resulting from abfraction, abrasion, attrition, erosion, or a combination of these factors. DH can develop due to dental diseases, as well as certain habits and environmental factors [21]. In this study, DH was most commonly observed in premolar teeth, with a prevalence of 38.6%. This result was consistent to findings from other studies in the literature [22, 23]. The increased prevalence of DH in premolar teeth may be attributed to the fact that abrasion is most commonly observed in these teeth [24].

It has been reported that DH generally has a higher prevalence in women [25]. Although there is no definitive evidence that gender is a risk factor for DH, Que et al. [26]. suggested that women’s increased sensitivity to pain might contribute to higher levels of DH. This may explain the fact that 62% of the study population was female.

In the treatment of DH, it has been stated that primary treatment strategies should focus on preventive measures aimed at eliminating the underlying factors [21]. Accordingly, participants in the study were instructed to avoid acidic foods and beverages and to brush their teeth using proper techniques without applying excessive force. Considering the higher prevalence of DH in premolar teeth and women, special attention and additional preventive measures may be necessary for these groups.

In 1997, Holland [1] published a guideline for the design and implementation of clinical studies on DH. In this study, as recommended in this guideline, we assessed DH across multiple regions such as incisors, canines, and premolars, and included individuals with varying levels of sensitivity. This study lasted 8 weeks, during which the pain intensity of individuals was evaluated using a cold air stimulus (SSS) and VAS. However, longer follow-up periods could provide more meaningful and comprehensive results in terms of controlling DH. For example, a study conducted by Kielbassa et al. [27] demonstrated that the effects of treatment continued over a 12-month observation period. Such long-term studies can provide more detailed information about the continuity and permanence of the treatment response. Therefore, longer observation periods should be considered in future studies for the evaluation of DH control.

DHEQ-15 is a tool specifically designed to better understand OHRQoL and assess the impact of DH on participants’ daily lives [28]. It plays a crucial role in understanding the impact of DH on restrictions in daily activities, changes in habits, interactions with others, emotional effects, and perceptions of health and/or age (identity). In a systematic review that included six clinical studies examining the impact of DH treatment on OHRQoL, DH treatment was found to improve OHRQoL by reducing DHEQ scores [29]. In an eight-week clinical study conducted in China, similar to this study, three different desensitizing toothpastes resulted in a significant decrease in DHEQ-15 scores compared to baseline [30]. In this study, baseline DHEQ-15 scores for all test groups of toothpastes were significantly higher than the DHEQ-15 scores at the 4th and 8th weeks. In the control group, no significant difference in DHEQ-15 scores was observed compared to baseline. Among the test toothpastes, CPP-ACP caused the most positive change in DHEQ-15 scores. This finding was consistent with the other results of the study.

Efforts to convert subjective feedback into objective data include both unidimensional and multidimensional pain measurement systems. In clinical research, the most commonly used unidimensional method for assessing the intensity of acute pain is the VAS [31]. In a study comparing the sensitivity and specificity of DH assessment scales, the SSS scale was identified as the most preferred scale for DH evaluation [20]. Based on these findings, the present study aimed to assess DH using the SSS scale and VAS. In a study investigating the effectiveness of two commercial toothpastes containing Potassium nitrate and Arginine for DH treatment, significant reductions were observed in VAS and SSS scores at weeks 1, 2, 4, and 8 [32]. In this study, the VAS and SSS scores for all test toothpaste groups were significantly higher at baseline compared to the scores at weeks 4 and 8. Among the test toothpastes, the most significant improvement in VAS and SSS scores was observed in the CPP-ACP groups. In the Control group, although a decrease was observed at the end of week 8, it was less pronounced compared to the test toothpastes. This result was consistent with findings from other clinical studies, which indicated that the fluoride toothpaste used in the control group had a minimal, yet positive, effect on the treatment of DH [33, 34]. This improvement was thought to stem from the increased resistance of dentin to acid demineralization and precipitation within the open dentinal tubules [35].

In this study, Arginine caused a significant decrease in DHEQ-15, SSS, and VAS scores starting from the 4th week, and the change at the end of the 8th week was greater than that observed in the control group for all three assessments. In a study involving 121 DH patients using an 8% Arginine-containing toothpaste for 8 weeks, significant reductions in SSS were observed at the 2nd, 4th, and 8th weeks [36]. Likewise, patients who received a single in-office treatment with Arginine desensitizing paste and then brushed twice daily with Arginine toothpaste for 24 weeks showed significant improvements in SSS in assessments conducted between weeks 8 and 24 [37]. In another study, Arginine was found to be the most effective among three commercial toothpastes containing fluoro-calcium-phospho-silicates, strontium acetate, and Arginine, showing the greatest reduction in VAS and SSS scores over a 6-week period [38].

In the present study, Arginine, together with Potassium nitrate, was one of the most effective toothpaste groups after CPP-ACP in reducing DHEQ-15, SSS and VAS scales, although Potassium nitrate ranked behind on the VAS scale by the end of the 8th week. Potassium nitrate’s mechanism, which involves blocking nerve stimuli in dentinal tubules, contributes to its desensitizing properties, differing from the action of other agents [39]. Supporting this, a study examining the effectiveness of a 3% Potassium nitrate mouthwash in DH treatment found significant improvements in DH in the Potassium nitrate group compared to a Control group using a different mouthwash [40]. Similarly, Hall et al. [41] showed that individuals using a 3% Potassium nitrate mouthwash, in addition to fluoride toothpaste, exhibited significantly greater reductions in DH at the 4th and 8th weeks compared to those using only fluoride toothpaste, as measured on the VAS and SSS scales.

In the literature, the effectiveness of toothpastes containing Arginine and Novamin in the treatment of DH has been compared, but a clear superiority between the two has not yet been determined. In a study comparing Arginine and Novamin, it was observed that while both toothpaste groups showed a reduction in sensitivity symptoms at the end of 15 days, the toothpaste containing Novamin was found to be more effective in reducing DH compared to the toothpaste containing Arginine [42]. In another study comparing the clinical effectiveness of toothpastes containing Arginine and Novamin, the toothpaste containing Arginine was found to be more effective than the toothpaste containing Novamin on the VAS and SSS scales [43]. In the current study, Novamin was found to be more effective than the control group in DH treatment across all three measurement methods, but, in parallel with the previous study, the change it induced was lower compared to that of Arginine group. Additionally, for Novamin, the changes in all parameters at the end of the 8th week compared to baseline were relatively lower than those observed with the other desensitizing agents, while the Propolis group showed similarly lower changes in the SSS and VAS assessments.

In modern dentistry, the use of Propolis as a desensitizing agent has garnered increasing interest in recent years through various studies. In a study investigating the effectiveness of a Propolis-containing toothpaste in DH treatment, significant reductions in DH were observed in both the 10% and 15% propolis extract groups compared to the control group, as measured by VAS and air stimulus tests [44]. In an in vitro study investigating the effectiveness of Propolis-based toothpaste in DH treatment, the Propolis-based toothpaste was found to be more effective in occluding dentinal tubules compared to the control group [9]. In a systematic review investigating the effectiveness of Propolis extract in DH treatment, all six clinical studies examined determined that Propolis was more effective and safe as a desensitizing agent compared to placebo [45]. In a clinical trial involving 120 patients, which compared the effectiveness of CPP-ACP and fluoride, Propolis, sodium fluoride, and placebo groups, the Propolis group was found to be the most effective in reducing DH [34]. In the present study, similarly, Propolis demonstrated greater effectiveness in DH treatment compared to the control group.

CPP-ACP has the potential to reduce DH by promoting the deposition of minerals that occlude dentinal tubules [45]. The effectiveness of CPP-ACP in DH treatment is supported by various clinical and laboratory studies, and this agent is widely used in dentistry as a desensitizing agent. In a study where CPP-ACP was used for the treatment of 104 teeth with DH over a 4-week period, significant improvement was observed in response to VAS and air stimulus compared to baseline levels [46]. In a 6-week study comparing the effectiveness of CPP-ACP and Potassium nitrate in 48 individuals with DH, although both products were effective on cervical dentine hypersensitivity, CPP-ACP was found to be significantly more effective than Potassium nitrate, as in the current study [47]. In a 21-day treatment of 73 teeth with DH, divided into three different treatment groups—CPP-ACP, Propolis extract, and sterile water—responses to air stimuli and VAS scores were recorded. Significant reductions in DH were observed in both the CPP-ACP and propolis treatment groups. Also, similar to the present study, CPP-ACP was found to be more effective in reducing DH compared to Propolis [48]. In the study by Ghiorghe et al. [49] the effectiveness of a remineralization agent containing CPP-ACP and fluoride and Novamin was evaluated for DH treatment using the SSS. According to this study, although both agents were effective in DH treatment by the 7th day compared to baseline, the remineralization agent containing CPP-ACP and fluoride was found to be more effective than the Novamin-containing toothpaste.

In this study, CPP-ACP and Potassium nitrate were used in accordance with the instructions and in combination with fluoride-containing toothpaste, as recommended by the literature [11, 41]. However, the Arginine, Novamin, and Propolis groups were not used in combination with any products for the same reasons. Although CPP-ACP, used as a two-product combination, was identified as the most effective agent in DH treatment, Potassium nitrate did not demonstrate the expected high effectiveness when evaluated in this context. Therefore, the idea that the difference in effectiveness was solely due to the addition of fluoride-containing toothpaste lost its validity. The fluoride-free Propolis toothpaste group demonstrated a similar effectiveness in the DHEQ-15 and VAS scales to Potassium nitrate used in combination with fluoride-containing toothpaste, and a higher effectiveness compared to the control group. In future studies, the addition of sodium fluoride to propolis-containing toothpastes could aim to develop more effective agents for DH treatment.

Among the limitations of the study were the presence of many factors that can influence pain measurement in patient-based sensitivity studies. The standard procedures used for testing products designed for DH treatment had not yet been sufficiently developed, making it difficult to compare the effectiveness of these products. Additionally, there was no control over patients’ oral hygiene status. Another limitation was the failure to identify factors that could contribute to DH, such as the consumption of acidic foods and drinks, improper brushing habits, and the evaluation of medically intrinsic or extrinsic erosive factors at the beginning of the study. The continuation of individual habits that may contribute to DH during or after the treatment process could lead to the reopening of dentinal tubules and, consequently, the reoccurrence of DH. Also, the treatment duration being limited to only 8 weeks has made it impossible to assess the long-term effects of the agents. This highlights the need for further long-term studies to assess the continuity of treatment response, which identify individual habits that may contribute to DH at the start of the study and control them throughout and after the treatment process.

## Conclusions

Within the limitations of this study, it can be concluded that the CPP-ACP group, which combined toothbrushing and inunction, was the most effective agent in reducing DH symptoms, as it demonstrated the greatest reduction in scores by the end of week 8 compared to baseline.

## Data Availability

The research data is available as an SPSS file with the corresponding author. If needed, the study data can be shared with the Clinical Oral Investigations journal.
